# Evaluation of MicroRNAs Regulating Anoikis Pathways and Its Therapeutic Potential

**DOI:** 10.1155/2015/716816

**Published:** 2015-10-26

**Authors:** Sharan Malagobadan, Noor Hasima Nagoor

**Affiliations:** ^1^Institute of Biological Sciences (Genetics & Molecular Biology), Faculty of Science, University of Malaya, 50603 Kuala Lumpur, Malaysia; ^2^Centre for Research in Biotechnology for Agriculture (CEBAR), University of Malaya, 50603 Kuala Lumpur, Malaysia

## Abstract

Dysregulation of microRNAs (miRNAs) has been implicated in almost every known survival mechanisms utilized by cancer cells. One of such mechanisms, anoikis resistance, plays a pivotal role in enabling metastasis by allowing cancer cells to circumvent cell death induced by lack of attachment. Understanding how miRNAs regulate the various anoikis pathways has become the research question of increasing number of studies published in the past years. Through these studies, a growing list of miRNAs has been identified to be important players in promoting either anoikis or resistance to anoikis. In this review, we will be focusing on these miRNAs and how the findings from those studies can contribute to novel therapeutic strategies against cancer progression. We will be examining miRNAs that have been found to promote anoikis sensitivity in numerous cancer types followed by miRNAs that inhibit anoikis. In addition, we will also be taking a look at major signaling pathways involved in the action of the each of these miRNAs to gain a better understanding on how miRNAs regulate anoikis.

## 1. Anoikis and Cancer

Attachment of a cell to the extracellular matrix (ECM) is vital for the proliferation and survival functions to be carried out efficiently. If a cell gets detached, apoptosis may be induced. Cell death triggered by lack of attachment to the ECM is defined as anoikis. This regulatory mechanism is especially important for epithelial cells that undergo a robust rate of renewal [1]. Apart from ensuring that cells are only viable with the appropriate contact with ECM, anoikis is also involved in limiting the progression of cancer. Since anoikis was first characterized by Frisch and Francis 11 years ago [2], increasing number of studies have uncovered the relationship between anoikis inhibition and cancer.

Although complex regulatory systems have been identified to be involved in anoikis mechanisms [3–5], anoikis mediated cell death can be grouped into two general pathways: intrinsic and extrinsic [6]. The intrinsic pathway involves caspase enzymes that are activated as a result of permeabilization of outer mitochondrial membrane by proapoptotic proteins, such as the members of B-cell lymphoma 2 (Bcl-2) family [7]. On the other hand, extrinsic pathway involves death receptors, such as the tumor necrosis factor (TNF) superfamily receptors, and the eventual activation of caspase-8. Both pathways are not biologically distinct, however, as it is known that the intrinsic pathway can also be induced by caspase-8, when it is activated by extrinsic pathway [8, 9].

The role of initiating anoikis comes down to proteins that mediate cellular interaction with the ECM and neighboring cells, such as integrins and E-cadherin. Integrins are receptors made up of two components, *α* and *β* subunits. In its ligated conformation, integrins can activate signaling pathways that promote survival such as the PI3K/Akt pathway [10] and promote apoptosis in its unligated conformation [9]. The apoptotic response by integrins have been documented and shown to be mediated through recruitment of caspase-8 [11, 12]. Meanwhile, E-cadherin is a transmembrane protein, with the cytoplasmic domain being bound to *β*-catenin, which then binds to *α*-catenin. As *α*-catenin can bind to the actin filaments, the complex as a whole plays an important role of connecting E-cadherin to the cytoskeleton, allowing it to regulate adhesion between cells [13, 14]. E-cadherin has also been shown to regulate anoikis through the PI3K/Akt and RAF/ERK pathways [15, 16]. For example, E-cadherin was found to be necessary for anoikis evasion in the absence of ECM in hepatocyte spheroids [17]. Furthermore, the loss of E-cadherin and gain of N-cadherin expression, the hallmark of epithelial-to-mesenchymal transition (EMT), has been repeatedly shown to promote inhibition of anoikis and subsequently enable metastasis [18, 19].

Acquisition of anoikis resistance is crucial in the progression of cancer cells to become metastatic. For example, anoikis resistance has been shown to be necessary for sphere formation. Sphere formation is a well-known capability of stem cells, although cancer cells have also been shown to undergo dedifferentiation to initiate sphere formation [20, 21]. With respect to tumorigenesis, sphere formation is an important characteristic of an aggressive cancer as it enables cancer cells to metastasize and initiate secondary tumor formation [22].

## 2. Anoikis and Cancer Associated MicroRNAs

MicroRNAs or miRNAs are short strands of RNA molecules, usually ranging between 17 and 27 nucleotides in size. These miRNA molecules play an integral role in regulating messenger RNAs (mRNA) after transcription. This regulatory mechanism involves the binding of miRNA to a target mRNA with complementary sequence, leading to the degradation of the mRNA [23]. By directly interacting with mRNA, miRNA is capable of silencing the expression of a target gene. The complexities of miRNA lie in the fact that a single miRNA can regulate multiple genes [24] and multiple miRNAs can regulate the same gene [25]. MiRNAs are well known to be dysregulated in various cancer types. This has led to an increasing amount of publications examining the role that these small sequences of nucleotide play in promoting and inhibiting cancer. As such, it comes as no surprise that numerous miRNAs have been found to play a pivotal role in disrupting various pathways regulating anoikis, to either confer anoikis resistance or anoikis sensitivity.

**Figure 1:**
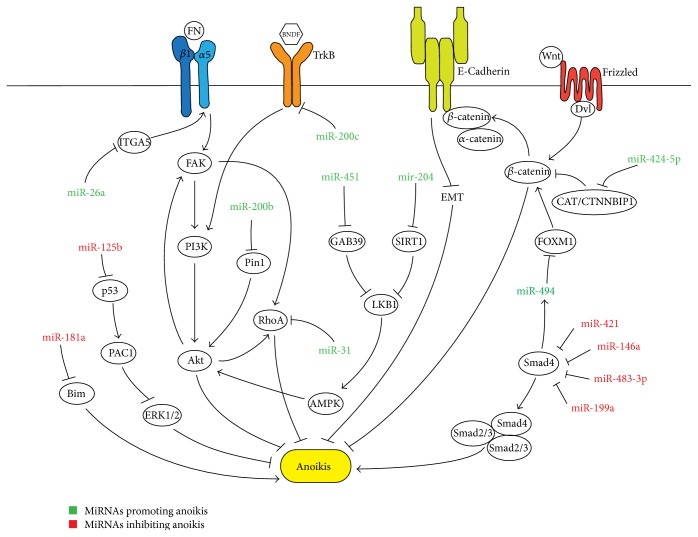
Schematic figure illustrating how miRNAs both positively and negatively regulate anoikis by targeting and manipulating key components of various signaling pathways.

In this review, such miRNAs (Figure 1) and how the findings from these studies can be utilized for therapeutic purposes are examined. We will first look at miRNAs that have been found to promote anoikis sensitivity in various cancer cell lines and the mechanisms through which it takes place. MiRNAs that play a role in inhibiting anoikis have also been identified and are reviewed as well. In addition, key pathways involved in the regulation of anoikis by miRNAs are highlighted to elucidate the underlying mechanisms.

## 3. MiRNAs Promoting Anoikis Sensitivity

The expression of miRNAs promoting anoikis is often downregulated in various cancer types, especially in variants that show anchorage independent growth. By performing various* in vitro* studies, the role played by such miRNAs in promoting anoikis has been demonstrated (Table 1(a)).

### 3.1. miR-204

In a study conducted by Zhang et al. (2013), downregulation of miR-204 was found to promote gastric cancer cell invasion by disrupting SIRT1-LKB1 pathway [26]. Liver kinase B1 (LKB1) is a tumor suppressor known to regulate the AMP-activated protein kinase (AMPK) family consisting of 13 kinases, which ultimately regulates a wide array of cellular processes such as cell growth, cell polarity, and energy metabolism [27]. However, a study done by Cheng et al. (2009) reported that LKB1 is also coupled to the p53-dependent anoikis [28]. Previous studies by the authors have shown sirtuin 1 (SIRT1) to be correlated to an aggressive metastatic phenotype in gastric cardiac carcinoma [26]. SIRT1, a class III histone deacetylase, exerts inhibitory effects against LKB1 by targeting it for degradation, subsequently promoting anoikis resistance and EMT. Luciferase reporter assay was able to confirm SIRT1 to be the direct target of miR-204 and overexpression of miR-204 elevated LKB1 expression and increased anoikis sensitivity in gastric cancer cells.

Another recent study done by Wang and colleagues reported findings that miR-204 was similarly downregulated in human posterior capsule opacification (POC) tissue [29]. Although not tumorigenic, POC is the result of normal attached lens epithelial cells (LEC) undergoing EMT due to the aggravation caused by cataract surgery. Interestingly, in this study Smad4 was found to be the target of miR-204, which is a part of the TGF-*β*/Smad signaling pathway, suggesting a complex regulatory role played by miR-204 in promoting anoikis and repressing EMT in epithelial cells.

### 3.2. miR-26a

MiR-26a has already been well established as a tumor suppressor miRNA in hepatocellular carcinoma (HCC), with roles of inhibiting tumor growth, metastasis [30], and angiogenesis [31]. Another recent study by Zhang et al. (2015) demonstrated how miR-26a specifically promoted anoikis in HCC. In their study, they found that miR-26a was downregulated, corresponding to an increased expression of integrin alpha-5 (ITGA5) in HCC [32]. ITGA5 forms a heterodimer complex with integrin beta-1 (ITGB1) to form the *α*5*β*1 integrin, which is capable of activating the focal adhesion kinase (FAK) [33] and suppressing anoikis. Overexpression studies in both* in vivo* and* in vitro* proved miR-26a increases anoikis sensitivity in HCC, consistent with its function of targeting ITGA5. In addition, overexpression of miR-26a also revealed a reduced Akt phosphorylation in tandem with reduced expression of ITGA5, suggesting that the PI3K/Akt pathway is also negatively regulated by this miR-26a. As this miRNA negatively regulates multiple targets that are involved in inhibiting anoikis, miR-26a makes an ideal candidate for therapeutic approach for liver cancer.

### 3.3. miR-31

Valastyan et al. (2009) demonstrated that overexpression of miR-31 in breast cancer cell lines returned sensitivity to anoikis mediated cell death, as much as 60% as was shown in MDA-MB-231 cell line [34]. Computational analysis revealed a list of miR-31 targets, most of them being genes regulating motility-related processes. From this list of genes, luciferase reporter assay and siRNA mediated silencing revealed RhoA and ITGA5 to be the two most prominent anoikis related genes targeted by miR-31. RhoA, a small G protein belonging to the Rho family, is regulated by the PI3K/Akt pathway. Inhibition of RhoA in melanoma cells has shown to increase rate of anoikis by inactivating FAK and reducing ITGA5 integrin expression in melanoma cells [35]. In addition to be regulated by RhoA, ITGA5 was also shown to be negatively regulated by miR-31, similar to miR-26a [32], as a mechanism to promote anoikis in a variety of breast cancer cell lines by Valastyan and colleagues.

Interestingly, despite being able to suppress metastasis* in vitro*,* in vivo* studies uncovered that ectopic expression of miR-31 resulted in increased growth of primary tumor in mice, highlighting the complexities of miRNA regulation. Similar characteristics have also been observed with other miRNAs playing multiple roles, each utilizing independent pathways [36, 37].

### 3.4. miR-451

Based on a previous study that confirmed miR-451 role in promoting anoikis [38], Tian and colleagues were able to identify the target of miR-451 to be CAB39, which is part of the LKB1-STRAD-MO25 complex [39]. This complex which is bound to LKB1 ensures that LKB1 is activated and localized in the cytoplasm [40], which in turn activates the P13K/Akt pathway. The relationship between the LKB1/AMPK and the PI3K/Akt pathways has already been established as well [41], where AMPK was found to activate Akt. Western blot on human glioma transfected with miR-451 mimics showed a reduced expression of multiple signaling factors upstream of Akt, such as LKB1, AMPK, and PI3K. These results suggest that miR-451 promotes sensitivity to anoikis in human glioma by inhibiting the expression of CAB39 and disrupting the P13K/Akt pathway.

In addition to glioma cells, miR-451 has also shown to promote anoikis sensitivity when overexpressed in nonsmall cell lung cancer (NSCLC) [42]. However, in this study, RAB14 was identified as a novel target of miR-451. Belonging to the Ras oncogene family, RAB14 inhibition through miR-451 overexpression resulted in a decreased phosphorylation of Akt, consequently promoting anoikis in NSCLC.

### 3.5. miR-200b

miR-200b belongs to the miR-200 family, which is well studied and established for their role in regulating E-cadherin. One of the miR-200 family members, miR-200b, was found to promote anoikis by negatively regulating Pin1 expression, which was confirmed using luciferase reporter assay [43]. Pin1 is crucial for the stability of activated ERK and Akt, as it binds and regulates the phosphorylated conformation of those signaling proteins [60, 61]. Consequently, elevated level of Pin1 is one of the mechanisms used by cancer cells to avoid anoikis through the activation of the ERK/Akt pathway. In the study by Zhang and colleagues, anoikis promoting effect of miR-200b was evident when overexpression of miR-200b resulted in increased sensitivity to anoikis in MDA-MB-231 breast cancer cell line, while coexpressing miR-200b with Pin1 negated that effect [43].

In addition to regulation of anoikis by miR-200b, the authors also investigated how miR-200b itself is regulated. Analysis on miR-200b promoter showed that it contained binding sites for polyomavirus enhancer activator 3 (PEA3) and ELK-1, both members of a larger E-twenty-six (Ets) family that have been previously implicated as upstream regulators of miRNAs [62, 63]. The authors performed siRNA mediated silencing and luciferase reporter assay which led to the conclusion that PEA3 was responsible for increasing the expression of miR-200b while ELK-1 was decreasing miR-200b expression by acting on its promoter.

### 3.6. miR-200c

miR-200c is another member of the miR-200 family that promotes anoikis. As with other miR-200 family members, miR-200c is also capable of restoring E-cadherin expression by targeting its transcriptional repressors. However, a study using a cell line that did not regain expression of E-cadherin showed that miR-200c was still inhibiting invasion and migration, suggesting that other pathways are involved which work independently of E-cadherin [44]. This led to a study by Howe et al. (2011) which investigated novel genes that are targeted by miR-200c. Microarray analysis and luciferase assay enabled them to identify a set of genes responsible for conferring mesenchymal and neuronal properties targeted by miR-200c [45]. Of these targets, TrkB was found to be responsible for conferring anoikis resistance in breast cancer cell lines. A receptor tyrosine kinase commonly found in neurons, TrkB is also being discovered in cancer cells owing to mutations or chromosomal rearrangements [64]. The role played by TrkB in cancer cells has also been studied, where TrkB was found to activate the PI3K/Akt pathway to enable survival in suspension [65]. In their studies, Howe and colleague found that both overexpression of miR-200c and downregulation of TrkB resulted in reversal of anoikis resistance [45].

Although this study shows miR-200c to be a tumor suppressor miRNA, further studies are required to understand the regulatory mechanisms across cancer types. This is because contrasting results were discovered in NSCLC, in a study which reported that elevated serum level of miR-200c correlated with shorter overall survival among patients [46].

### 3.7. miR-424-5p

Through microarray profiling performed on anoikis resistant variant of the HCC cell line, miR-424-5p was found to be significantly downregulated [47]. This study by Zhang and colleagues further emphasized the role played by E-cadherin on anoikis regulation. This is because ICAT/CTNNBIP1, a *β*-catenin inhibitor, was found to be a direct target of miR-424-5p. As a result, miR-424-5p is able to maintain the level of E-cadherin/*β*-catenin complex to prevent EMT from occurring. In fact, they were also able to demonstrate a reversal of EMT in the HCC cell line by increasing the expression of miR-424-5p. The role of *β*-catenin in anoikis is also attributable to the activation of the Wnt pathway, in which *β*-catenin interacts with transcription factor lymphoid enhancer-binding factor 1 (LEF-1) to regulate gene expression and subsequently inhibit anoikis [66–68]. Furthermore, the study was advanced to clinical investigation, allowing them to draw a correlation between decreased expression of miR-424-5p with late clinical stage in liver cancer progression. Together, the results from this study highlight the therapeutic potential of miR-424-5p in suppressing the malignant phenotype of metastatic liver cancer.

### 3.8. miR-494

MiR-494 was chosen by Li and colleagues for a study in pancreatic ductal adenocarcinoma (PDAC) as it was found to target FOXM1 [48]. FOXM1 is a transcriptional factor known to interact with *β*-catenin and enable its translocation in glioma cells [69]. As *β*-catenin is a part of the Wnt signaling pathway, its nuclear translocation aided by FOXM1 will result in activation of various tumor promoting genes. As such, by targeting FOXM1, miR-494 was found to inhibit transcriptional activity of *β*-catenin and interrupt the Wnt pathway [48]. Overexpression of miR-494 in PDAC also suppressed anoikis resistance as was evident through the colony formation assay.

The authors also investigated the reason for downregulation of miR-494 in PDAC and found that loss of Smad4 expression in PDAC resulted in a defect in the TGF-*β* signaling, which ultimately caused a downregulated expression of miR-494. Overall, reduced expression of miR-494 serves as an evidence for a defective TGF-*β* signaling pathway and will be useful to determine the malignant nature of pancreatic cancer.

## 4. MiRNAs Promoting Anoikis Resistance

Unsurprisingly, miRNAs that inhibit anoikis and confer resistance have been found to be upregulated in cancer cells. These miRNAs play oncogenic role by enabling survival of cancer cells in suspension, thus enabling metastasis to occur. The targets and pathways regulated by these miRNAs have been analyzed and published (Table 1(b)).

### 4.1. miR-125b

Based on studies by Yu and colleagues using mesenchymal stem cells, miR-125b was shown to protect cells from anoikis induced cell death [49]. This study was done by subjecting mesenchymal stem cells to growth in suspension and screening for miRNA expression level. Mir-125b, which was expressed in high level in suspended variant of the human mesenchymal stem cells (hMSC), was found to enable survival for up to 7 days in suspension. The mechanisms by which miR-125b conferred anoikis resistance which involve ERK signaling were also demonstrated by the author.

By targeting and suppressing p53, miRNA-125b was able to increase ERK phosphorylation in the mesenchymal stem cells [70]. This study highlights another major component involved in anoikis, p53, a tumor suppressor which is repeatedly shown to promote sensitivity to anoikis [15] and loss of its expression is often correlated to anoikis resistance [71, 72].

The authors performed knockdown of miR-125b to study its effect, which increased rate of anoikis in hMSC in suspension. However, whether or not miR-125b has similar effect on cancer cells is yet to be determined, as further studies showed that miR-125b did not protect human umbilical endothelial cells (HUVEC) from anoikis, suggesting that this regulation of anoikis may vary greatly across cell types [49].

### 4.2. miR-181a

A study by Wang et al. (2011) looked at an interesting feature in breast cancer cell lines similar to stem cells, which is to form spheres termed mammospheres when in suspension. In their studies, upregulation of miR-181 by TGF-*β* was found to be responsible for the regulation of sphere formation [73]. However, how miR-181 was regulating anoikis to enable sphere formation was not identified. This was carried out in a recent study in breast cancer cells. Taylor and colleagues looked at how miR-181a was contributing to anoikis resistance and discovered that elevated level of miR-181a was enabling higher survival rate for breast cancer cells in suspension [50]. Transfection of miR-181a mimics in normal murine mammary gland (NMuMG) cells and 4T1 breast carcinoma rendered the cells less sensitive to anoikis. Western blot and computational analysis showed Bim, a proapoptotic protein, to be the anoikis related target for miR-181a. They also made an interesting discovery when they found out that Bim mRNA itself was not reduced by increasing level of miR-181a expression, suggesting that the inhibition of Bim mRNA takes place at the translational level instead of the usual degradation of mRNA. The authors also verified that the regulation of Bim and anoikis takes place independently of the ERK/Akt pathway, as miR-181a activity was not affected by inhibition of Erk1/2 or Akt.

### 4.3. miR-145

In a recent study, Derouet and colleagues investigated miR-145 when they found that its expression was increased in esophageal adenocarcinoma (EAC) upon chemoradiation treatment. Interestingly, overexpression of miR-145 in EAC and esophageal squamous cell carcinoma (ESCC) cell lines showed contrasting results, with miR-145 promoting anoikis resistance and cell growth in EAC while producing the opposite results in ESCC [51]. The authors also performed cell adhesion assay and found that the expression of miR-145 increased the cellular adhesion to fibronectin. In this study, the target gene for miR-145 was not determined or validated. However, since fibronectin is known to interact with *α*5*β*1 integrin to promote metastasis [74], the authors suggest that the antianoikis effect in EAC could be due to upregulation of *α*5*β*1 integrin, based on results from the cell adhesion assay.

### 4.4. miR-421

A study by Hao et al. (2011) comparing pancreatic cancer tissues with normal adjacent tissues showed that miR-421 was overexpressed in the cancer tissues [52]. Developing on this result, they conducted another study to determine role of miR-421 in pancreatic cancer. Based on target prediction analysis and luciferase reporter assay, Smad4 was determined to be the target for miR-421. Predictably, overexpression of miR-421 increased anoikis resistance, based on the colony formation assay in which the overexpression increased colony size and numbers in anchorage independent condition. They also performed real-time PCR to investigate the expression of transcriptional targets regulated by Smad4 and found that expression of* p21* and* p15*, targets of TGF-*β* and* Id3* and* Id4*, and targets of bone morphogenetic protein (BMP) were inhibited as expected, illustrating the mechanism of how Smad4 targeting by miR-421 affects the cancer cell phenotype.

### 4.5. miR-146a

Interestingly, miR-146a has shown diverse expression pattern in different cancer cell types, such as being upregulated in breast cancer [75] and cervical cancer [76] while being downregulated in papillary thyroid carcinoma [53]. MiR-146a has been previously found to target Smad4 in promyelocytic leukemia cell line in a study by Zhong et al. (2010) [54]. In regard to anoikis regulation, Xiao and colleagues investigated if miR-146a plays a similar role in gastric cancer [55]. Based on luciferase reporter assay, they were able to confirm the role miR-146a in suppressing Smad4 expression in multiple gastric cancer cell lines [55]. They went on further to find the expression of miR-146a and Smad4 mRNA to be inversely correlated, supporting the luciferase reporter assay result.

However, another study that analyzed gastric cancer tissue samples found contrasting results, in which the expression of miR-146a was found to be downregulated in most of the samples [56]. This discrepancy regarding contrasting expression pattern of miR-146a needs to be addressed before it can be applied in any therapeutic approach.

### 4.6. miR-483-3p

MiRNA-array differential analysis performed by Hao and colleagues revealed miR-483-3p to be upregulated in pancreatic cancer tissues. The authors narrowed down target to be Smad4 and confirmed using luciferase reporter assay [57]. Anoikis specific studies were not performed, although overexpression studies showed increased colony formation, which requires the cells to have an increased resistance to anoikis.

Another study was done to determine the effects of miR-483-3p by Bertero et al. (2011) in keratinocytes. This study found the miRNA to regulate multiple genes related to cell growth and overexpression of miR-483-3p in keratinocytes restricted both migration and proliferation [58]. Taken together, these studies suggest a complex role played by miR-483-3p that may help to suppress multiple oncogenic aspects of cancer cells, making it a desirable candidate for miRNA based therapy.

### 4.7. mir-199a

For this study, Zhang et al. (2012) employed the approach of working backwards, by first selecting the target gene as* SMAD4* followed by a functional screening to find candidate miRNAs that target this gene [59]. Using an expression library of 388 human miRNAs, they performed luciferase reporter assay to screen for miRNAs that target Smad4 3′UTR. This result combined the bioinformatics analysis to narrow down the list to three miRNAs that elicited a significant reduction in Smad4 expression. One of these miRNAs, miR-199a, was chosen for further studies. Based on overexpression studies, they found that miR-199a promoted anoikis resistance in gastric cancer cells by encouraging anchorage independent growth.

Interestingly, another study that looked at TGF-*β*1 signaling pathway and miR-199a found the miRNA to be upregulated in response to TGF-*β*1 in human primary pulmonary artery smooth muscle cells [77]. However, Zhang and colleagues were not able to replicate similar response in gastric and liver cancer cell lines, SNU-16 and HEPG2, respectively, suggesting that the regulation of TGF-*β*1 pathway by miRNAs is still unclear and differs with cell lines [59].

## 5. MiRNAs and Signaling Pathways Regulating Anoikis

Although anoikis is a complex regulatory mechanism spanning multiple signaling pathways and mechanisms [8], analysis of dysregulated miRNAs in cancer cells have revealed several key pathways that work to either promote or inhibit anoikis. While different genes are targeted by these miRNAs, it is clear that the targets are often components of the same pathways (Table 1), highlighting the important role played by these pathways.

### 5.1. PI3K/AKT Pathway and miRNAs Promoting Anoikis

It is evident from the miRNAs reviewed here (Table 1(a)) that the PI3K/Akt pathway is the main target for majority of miRNAs promoting anoikis sensitivity. PI3K/Akt pathway is a prosurvival signaling pathway that plays a major role in various cellular functions such as proliferation, invasion, and angiogenesis. While it has been well established that the PI3K/AKT pathway components are often mutated to enable abnormal activation in various cancer types [78, 79], studies on miRNAs now show that cancer cells also downregulate miRNAs that targets this pathway as a strategy to evade anoikis. When such miRNAs (Table 1(a)) are overexpressed, PI3K/Akt pathway is disrupted and cancer cells exhibit increased sensitivity towards anoikis. Since PI3K/Akt pathway is well known to promote proliferation of various cancer types, targeting this pathway has become a desirable approach in cancer treatment, with extensive studies being done on this pathway in cancer cells. As a result, several drugs targeting components of this pathway are currently undergoing clinical trials [80, 81].

### 5.2. TGF-*β*/SMAD Pathway and miRNAs Inhibiting Anoikis

A tumor suppressor, Smad4 protein acts as an effector signaling molecule that works downstream of the TGF-*β* superfamily signaling [82]. Other components of the Smad family are functionally split into two pathways, with Smad2 and Smad3 in the TGF-*β*/activin pathway and Smad1, Smad5, and Smad8 in the BMP pathway. When these Smad proteins are phosphorylated by receptors, a heteromeric complex is formed with Smad4 as a partner, enabling the translocation of the complex to the nucleus for either direct interaction with DNA or with other transcription factors [83, 84]. Overall, the TGF-*β*/Smad pathways govern a plethora of functionalities including cell growth, survival, and differentiation [85].

A study done by Ramachandra and colleagues provided a better understanding regarding one of the mechanisms by which Smad4 regulates anoikis sensitivity in cancer cells [86]. By overexpressing Smad4, *β*1 integrin expression was found to be increased. This is consistent with another study which demonstrated that *β*1 integrin expression is augmented by the TGF-*β* pathways [87]. In its unligated form, which results from detachment from the ECM, *β*1 integrin actively promotes anoikis through caspase-8 mediated cell death [12]. With an important role in maintaining anoikis, Smad4 is often defective in cancer by means of mutation or deletion [82]. However, increasing number of studies are now showing that the TGF-*β*/Smad pathway is also dysregulated by many oncogenic miRNAs as a way to inhibit anoikis and initiate the metastatic cascade as presented in this review (Table 1(b)).

## 6. Current miRNA Based Therapeutics

MiRNA based cancer therapy has entered a very important stage in development, with several miRNAs and miRNA inhibitors being advanced past preclinical stages by pharmaceutical companies [88]. This development is instrumental to the study of miRNA as it has the consequence of proving that miRNAs' therapeutic potential can be translated from* in vitro* cancer model to clinical application in patients. An example of such miRNA based drug is miRNA-122 inhibitor that is capable of treating Hepatitis C infection. Interestingly, two separate companies are working on different miR-122 inhibitors, with Santaris Pharma currently at Phase II stage and Regulus Therapeutics at Phase I stage of clinical trial [89].

As for cancer therapy, one promising miRNA based drug, MRX34, is currently in Phase I stage [89]. MRX34 is a mimic of miR-34a, a well-known tumor suppressor miRNA in various cancer types [90]. By adopting a similar approach, anoikis related miRNAs can be great candidates for cancer treatment. Since anoikis is an early barrier for cancer cells to become metastatic, targeting anoikis related miRNAs will enable the suppression of cancer cells from becoming more aggressive. In addition, identifying individual miRNAs that regulate multiple cancer cell phenotypes such as anoikis resistance and metastasis will allow the development of a more potent miRNA based drug.

However, the application of anoikis related miRNAs in cancer therapy need not be restricted to treatment alone. As shown by multiple studies, elevated serum level of oncogenic miRNAs has been evident in cancer patients. Based on the growing body of knowledge on miRNAs regulating anoikis, a personalized approach to cancer therapy can be designed, where patients exhibiting increased expression of miRNAs inhibiting anoikis can be selected for a targeted treatment using inhibitors of those miRNAs.

## 7. Conclusion

Anoikis regulation by miRNA has proven to be a complicated yet important topic, with an increasing number of miRNAs found to play critical role in both promoting and inhibiting anoikis. From the evaluation of miRNAs involved in modulating anoikis, it has become evident that anoikis promoting miRNAs make for attractive candidates for anticancer therapy, as it has shown to be capable of not only curbing metastasis before it happens, but also reversing the malignant phenotype of an aggressive cancer. Furthermore, it is also apparent how some of the same signaling pathways have been repeatedly shown to be targets for various anoikis related miRNAs, such as the TGF-*β*/Smad and PI3K/Akt pathways, thus emphasizing the need to focus on these pathways in engineering cancer treatments.

While miRNAs promoting anoikis can be useful in therapeutic approach, miRNAs inhibiting anoikis can also play useful role through a novel prognosis method in cancer detection, as elevated levels of miRNAs conferring anoikis resistance have been detected in serum, showing high correlation with survival rate of patients. However, before miRNAs can be developed into viable anticancer treatment, the multiple functions played by miRNAs and the contrasting roles across different cell types need to be addressed.

In addition, another key issue that requires attention is the challenge of delivering known tumor suppressor miRNAs to cancer cells. If these issues are addressed accordingly, miRNAs regulating anoikis will greatly contribute to effective cancer prognosis and potential development of anticancer drugs.

## Figures and Tables

**(a):** 

MiRNA	Cancer type	Target	Signalling pathway	Reference
miR-204	Gastric	SIRT1	SIRT1-LKB1, PI3K/AKT	[26, 29]
miR-26a	Liver	ITGA5	PI3K/AKT	[30–32]
miR-31	Breast	RhoA, ITGA5	PI3K/AKT	[34]
miR-451	Glioma Nonsmall cell lung cancer	CAB39 Rab14	PI3K/AKT	[38, 39] [42]
miR-200b	Breast	Pin1	PI3K/AKT	[43]
miR-200c	Breast	TrkB	PI3K/AKT	[44–46]
miR-424-5p	Liver	CAT/CTNNBIP1	Wnt	[47]
miR-494	Pancreatic	FOXM1	Wnt	[48]

**(b):** 

MiRNA	Cancer type	Target	Signalling pathway	Reference
miR-125b	—	p53	MAP/ERK	[49]
miR-181a	Breast	Bim	—	[50]
miR-145	Esophageal	—	—	[51]
miR-421	Pancreatic	Smad4	TGF-*β*/SMAD	[52]
miR-146a	Leukemia	Smad4	TGF-*β*/SMAD	[53–56]
miR-483-3p	Pancreatic	Smad4	TGF-*β*/SMAD	[57, 58]
miR-199a	Gastric	Smad4	TGF-*β*/SMAD	[59]

## Abbreviations

AKT: Protein kinase B
AMPK: AMP-activated protein kinase
Bcl-2: B-cell lymphoma 2
BIM: Bcl-2-like protein
BMP: Bone morphogenetic protein
BNDF: Brain derived
CAB39: Calcium binding protein 39
CAT/CTNNBIP1: Catenin, beta interacting protein 1
Dvl: Dishevelled
EAC: Esophageal adenocarcinoma
ECM: Extracellular matrix
ELK-1: Member of E-twenty-six family
EMT: Epithelial-to-mesenchymal transition
ERK: Extracellular regulated MAP kinase
ESCC: Esophageal squamous cell carcinoma
FAK: Focal adhesion kinase
FN: Fibronectin
FOXM1: Forkhead box M1
HCC: Hepatocellular carcinoma
hMSC: Human mesenchymal stem cells
ITGA5: Integrin alpha-5
ITGB1: Integrin beta-1
LEC: Lens epithelial cells
LEF-1: Lymphoid enhancer-binding factor 1
LKB1: Liver kinase B1
MAP: Mitogen activated kinase-like protein
MiRNA: MicroRNA
mRNA: Messenger RNA
NSCLC: Nonsmall cell lung cancer
P53: Transformation related protein 53
PAC1: First procaspase activating compound
PDAC: Pancreatic ductal adenocarcinoma
PEA3: Polyomavirus enhancer activator 3
PI3K: Phosphatidylinositol 3-kinase
Pin1: Peptidylprolyl cis/trans isomerase, NIMA-interacting 1
POC: Posterior capsule opacification
Rab14: Member RAS oncogene family
RhoA: Ras homolog family member A
siRNA: Small interfering RNA
SIRT1: Sirtuin 1
TGF-*β*: Transforming growth factor beta
TNF: Tumor necrosis factor
TrkB: Tyrosine kinase receptor, type 2
Wnt: Wingless-type MMTV integration site family.
